# Investigating the Impact of Transportation Air/Noise Pollution on Mental Health and Implications for Healthy Aging

**DOI:** 10.1093/geroni/igaf122.099

**Published:** 2025-12-31

**Authors:** Dialechti Tsimpida, Anastasia Tsakiridi, Emily Oliver

**Affiliations:** University of Southampton, Southampton, England, United Kingdom; University of Southampton, Southampton, England, United Kingdom; Newcastle University, Newcastle upon Tyne, United Kingdom

## Abstract

Mental ill health is shaped by both social and environmental factors, yet the combined effects of air and noise pollution on mental health, particularly among older adults, remain largely underexplored. Disadvantaged communities are often disproportionately exposed to these stressors, amplifying their vulnerability. This study investigated the independent and combined effects of transportation-related air and noise pollution on depression across two Integrated Care Systems (ICS) in northern and southern England. Using data from the 2022 Quality and Outcomes Framework, we calculated depression prevalence for small areas within the Hampshire and Isle of Wight (HIOW) and North East and North Cumbria (NENC) ICS. Transportation-related nitrogen oxides and noise mapping data were used to assess transportation air and noise pollution levels, while neighborhood deprivation was measured using all domains of the English Index of Multiple Deprivation. Depression prevalence was derived from routinely collected publicly available mental health data. Generalized Structural Equation Spatial Modeling was employed to explore mediation effects between environmental stressors, socioeconomic deprivation, and depression at a small area level. In HIOW’s rural areas, noise exposure indirectly affected depression, accounting for 18.64% of the total effect of crime on depression. In NENC’s urban areas, older adults with higher nighttime noise exposure were more vulnerable to the mental health impacts of income deprivation. Combined air and noise pollution further exacerbated depression in older adults [indirect effect -5.0623 (p < 0.000001)]. These findings highlight the need for targeted, place-based public health strategies to reduce mental health inequalities and promote healthy aging in disadvantaged communities.

